# The browning and re-browning of lakes: Divergent lake-water organic carbon trends linked to acid deposition and climate change

**DOI:** 10.1038/s41598-019-52912-0

**Published:** 2019-11-13

**Authors:** Carsten Meyer-Jacob, Neal Michelutti, Andrew M. Paterson, Brian F. Cumming, Wendel (Bill) Keller, John P. Smol

**Affiliations:** 1 Paleoecological Environmental Assessment and Research Laboratory (PEARL), Department of Biology, Queen’s University, Kingston, ON K7L 3J9 Canada; 2 Department of Ecology and Environmental Science, Umeå University, 90187 Umeå, Sweden; 3 Dorset Environmental Science Centre, Ontario Ministry of the Environment, Conservation and Parks, Dorset, ON P0A 1E0 Canada; 4 Cooperative Freshwater Ecology Unit, Vale Living with Lakes Centre, Laurentian University, Sudbury, ON P3E 2C6 Canada

**Keywords:** Carbon cycle, Environmental monitoring, Geochemistry, Limnology

## Abstract

Dissolved organic carbon (DOC) concentrations and water colour are increasing in many inland waters across northern Europe and northeastern North America. This inland-water “browning” has profound physical, chemical and biological repercussions for aquatic ecosystems affecting water quality, biological community structures and aquatic productivity. Potential drivers of this “browning” trend are complex and include reductions in atmospheric acid deposition, changes in land use/cover, increased nitrogen deposition and climate change. However, because of the overlapping impacts of these stressors, their relative contributions to DOC dynamics remain unclear, and without appropriate long-term monitoring data, it has not been possible to determine whether the ongoing “browning” is unprecedented or simply a “re-browning” to pre-industrial DOC levels. Here, we demonstrate the long-term impacts of acid deposition and climate change on lake-water DOC concentrations in low and high acid-deposition areas using infrared spectroscopic techniques on ~200-year-long lake-sediment records from central Canada. We show that acid deposition suppressed naturally higher DOC concentrations during the 20th century, but that a “re-browning” of lakes is now occurring with emissions reductions in formerly high deposition areas. In contrast, in low deposition areas, climate change is forcing lakes towards new ecological states, as lake-water DOC concentrations now often exceed pre-industrial levels.

## Introduction

Monitoring programs across northern Europe and northeastern North America have recorded a marked “browning” (also referred to as “brownification”) of inland waters in recent decades, caused by increasing dissolved organic carbon (DOC) concentrations^1,2^. The DOC and associated water-colour increases affect water quality, increase costs for drinking water treatment^3^, and may alter the overall functioning of aquatic ecosystems because DOC concentrations influence physical and chemical water properties, and thus the structure and productivity of biological communities. For example, DOC affects light attenuation, oxygen and nutrient availability, and consequently the development of thermal stratification and hypoxia as well as species distributions and habitat availability^4,5^. Examples of chemical and biological repercussions of the current browning trend include reduced fish growth^6,7^, more favourable conditions for toxin-producing cyanobacteria blooms^8^, reduced potential for the inactivation of pathogens by solar ultraviolet radiation^9^, and increased contaminant transport^10,11^.

While several stressors, such as changes in land use/cover^12–14^, increased nitrogen (N) deposition^15,16^ and climate change^17,18^, can influence lake-water DOC levels, an increasing number of studies have attributed the DOC increase to the recovery of soils from atmospheric acid deposition, which enhances DOC solubility and thus its mobility from terrestrial to aquatic environments. For example, water-monitoring data have shown strong temporal correlations between changes in water chemistry resulting from reduced acid deposition (declining sulphate and ionic strength, increasing pH) and increasing DOC concentrations^1,19^, and experimental studies have demonstrated reduced DOC mobility in soils with increasing acidity^20,21^. However, the concurrent impacts of the different stressors complicate identifying their individual contributions to changes in DOC, and the lack of long-term data beyond traditional monitoring windows (a few decades at best) leaves it unclear whether the ongoing browning is unprecedented or predominantly a “re-browning” to natural pre-industrial levels.

**Figure 1:**
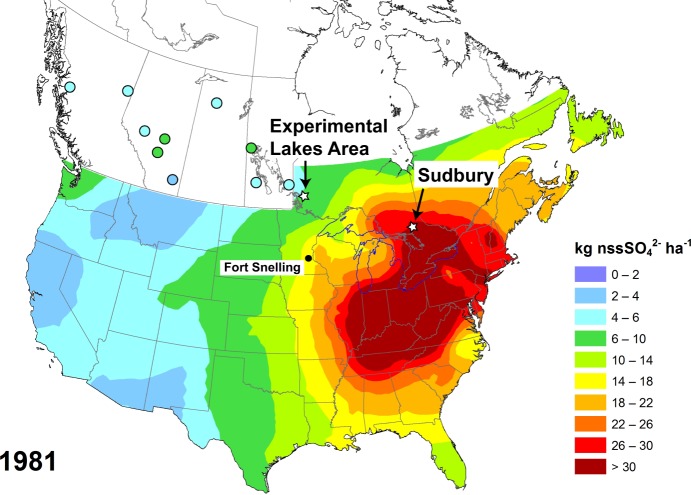
Annual atmospheric non-sea-salt (nss) sulphate (SO_4_^2−^) deposition (kg ha^−1^) across the U.S.A. and Canada in 1981^57^. Coloured circles refer to discrete deposition measurements. Stars indicate study regions, while the black dot represents the location of Fort Snelling, Minnesota, which has a temperature record reaching back to 1820.

**Figure 2:**
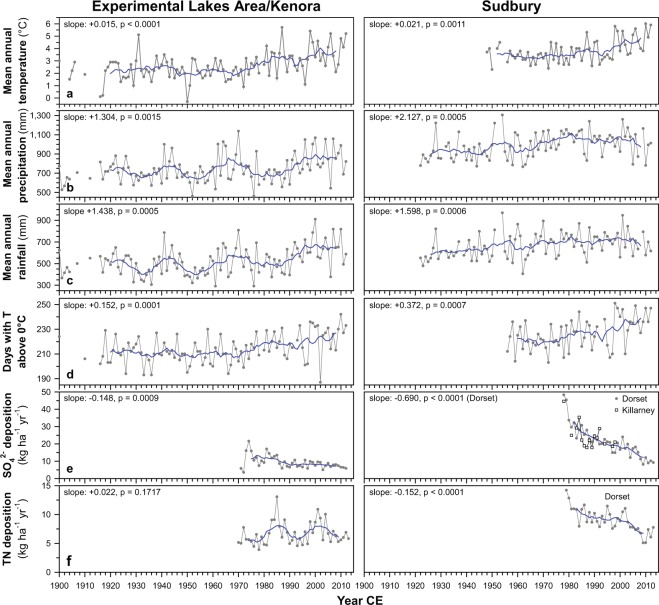
Climate and atmospheric deposition trends for Kenora, ~50 km east of the Experimental Lakes Area (ELA), and Sudbury, Ontario. Grey line plots indicate annual means, while blue lines represent 9-year running means. Slopes (annual change) and significance levels resulting from trend analyses are given for each variable. (**a**) Mean annual air temperature. (**b**) Mean annual precipitation. (**c**) Mean annual rainfall. (**d**) Days with temperatures above 0 °C. (**e**) Annual atmospheric sulphate (SO_4_^2−^) deposition for the ELA (1971–2012), Killarney (~60 km southwest of Sudbury; 1978–1998)^22^ and Dorset, Ontario (~220 km southeast of Sudbury; 1978–2013). (**f**) Atmospheric Total Nitrogen (TN) deposition for the ELA (1970–2013)^39^ and Dorset, Ontario (~220 km southeast of Sudbury; 1979–2013).

To determine the specific influence of acid deposition on lake-water DOC concentrations, we inferred long-term DOC trends from lake-sediment records over the past ~200 years, and compared these trends in lakes from historically low and high acid-deposition regions of central Canada. The Experimental Lakes Area (ELA) in northwestern Ontario received relatively low acid inputs (8.1 kg SO_4_^2−^ ha^−1^ yr^−1^ in 1990–1998), whereas the Sudbury region in northeastern Ontario was heavily affected by acid deposition during the 20^th^ century (>22.7 kg SO_4_^2−^ ha^−1^ yr^−1^ in 1990–1998) (Figs 1 and 2)^22^. Both regions are located in the Boreal Shield ecozone, and are characterized by northern mixed forests underlain by Precambrian Shield bedrock and glacial deposits. The study lakes in Sudbury and the ELA are oligotrophic (total phosphorus: 2.2–10.3 µg L^−1^), acidic to slightly alkaline (pH: 5.5–7.6), and small to medium-sized (lake area: 5–795 ha; Z_max_: 5–40 m; Z_mean_: 2–19 m; Supplementary Table S1). Annual temperature and precipitation averages (Canadian Climate Normals for 1981–2010) are 3.1 °C and 715 mm for Kenora, Ontario, ~50 km east of the ELA, and 4.1 °C and 903 mm for Sudbury. Smelting operations in Sudbury once represented one of the largest global point sources of atmospheric SO_2_ emissions, releasing up to 2,500 kt yr^−1^ during the 1960s. Acid deposition rates during 1960s peak emissions – and prior to the change from low- to high-level smoke stacks in 1972 – are unknown, but measurements from 1971 showed that sites in direct proximity to Sudbury smelters received up to 55 kg SO_4_^2−^ ha^−1^ in one month alone^23^. As a consequence, many lakes acidified, and vegetation and soils near smelting sites were severely altered^24,25^. In contrast to their different acid deposition histories, both regions have experienced a similar warming trend of 1.4–1.7 °C since the early 1900s (Fig. 2).

Sediment archives allow researchers to place recent environmental changes into a long-term perspective. Importantly, the few decades of overlap between monitoring and sediment records allow us to make quantitative comparisons between measured and sediment-inferred trends. Recent advances in analytical techniques now permit the inference of past DOC trends based on sediment visible-near infrared (VNIR) spectroscopy, a fast, inexpensive and non-destructive technique. Utilizing a calibration model between VNIR spectra of lake-surface sediments and corresponding surface-water DOC concentrations^26^, the technique allows the reconstruction of long-term DOC trends preserved in sediments on the scales of decades to millennia. In previous studies, VNIR inferred trends have successfully matched trends of water-chemistry monitoring data^12,26^, and have, for example, shown the impact of early land use^12,27^ and acid deposition^28,29^ on lake-water DOC in Sweden. However, while acid deposition could be identified as an important factor contributing to 20th-century DOC dynamics, centuries of anthropogenic landscape alterations in Scandinavia and their legacy effects on DOC dynamics confound identifying its individual contribution^12,14^. In contrast to Europe, extensive and widespread land alteration did not occur in North America before the arrival of European settlers.

We reconstructed past lake-water DOC trends using high-resolution ^210^Pb-dated sediment cores from 16 lakes, with eight each from Sudbury and the ELA, to identify regional DOC trends for the past ~200 years, and to determine: a) to what extent and on what timescale DOC levels have declined in response to acid deposition; b) to what degree DOC concentrations have recovered since sulphur emissions reductions; and c) how DOC levels might change in the future during continuing recovery, and in response to climate change-driven warming temperatures and changes in precipitation.

## Results and Discussion

Water-chemistry monitoring data show the recovery of Sudbury lakes from acid deposition, with increasing pH in acid-sensitive lakes and declining SO_4_^2−^ levels in all study lakes since the beginning of monitoring in the 1970s/1980s (Supplementary Figs S1 and S2). The low acid-deposition rates in the ELA are reflected by stable pH levels and considerably lower, yet also declining SO_4_^2−^ concentrations (decline from 10–30 to 5–15 mg L^−1^ in Sudbury vs. declines from ~4 to ~2 mg L^−1^ in the ELA; Supplementary Figs S1 and S2). In Sudbury lakes, DOC concentrations have increased by 1.5–5.5% yr^−1^ (0.03–0.1 mg L^−1^ yr^−1^) since the beginning of monitoring, while, in ELA lakes, DOC levels have only increased slightly by 0.1–0.5% yr^−1^ (~0.01 mg L^−1^ yr^−1^) (Supplementary Fig. S3).

**Figure 3:**
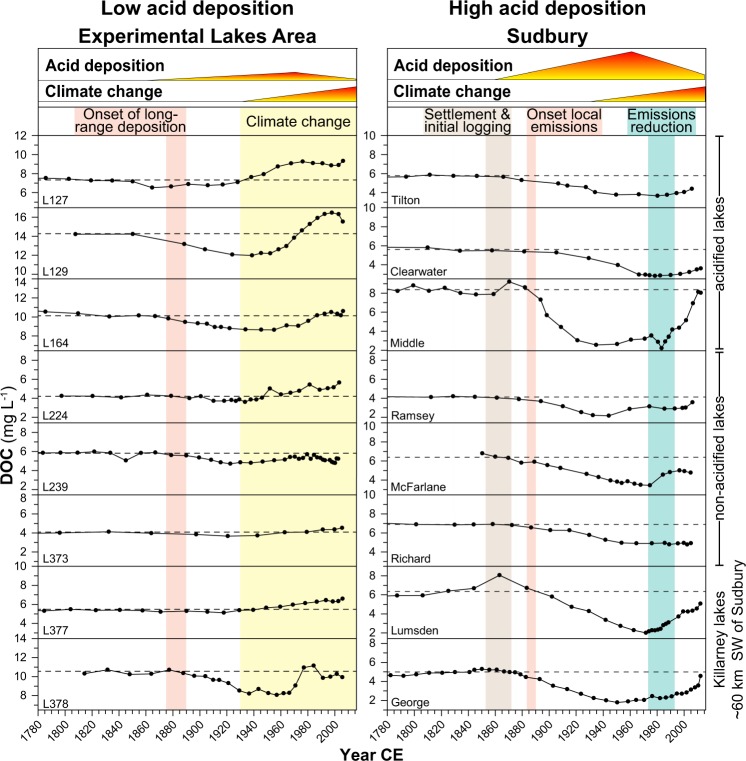
Sediment-inferred lake-water dissolved organic carbon (DOC) trends for the Experimental Lakes Area (ELA) and Sudbury over the last ~200 years.

**Figure 4:**
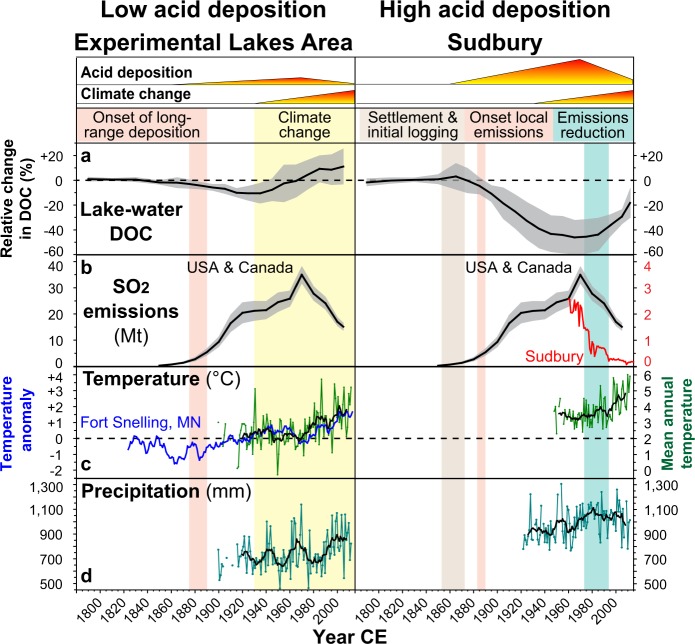
Regional lake-water dissolved organic carbon (DOC) trends for the Experimental Lakes Area (ELA) and Sudbury. (**a**) Relative change in sediment-inferred DOC concentrations (black) compared to the mean pre-industrial (1780–1850) levels (dashed). Grey envelope indicates the standard deviation from the mean based on eight lake-sediment records for each region. (**b**) Sulphur dioxide (SO_2_) emissions for the U.S.A. and Canada^30^ (black) and for Sudbury (red) in mega tonnes (Mt). (**c**) Mean annual air temperature (green, 9-year running mean in black) for Kenora, ~50 km east of the ELA, and Sudbury compared to the mean annual temperature anomaly for Fort Snelling, Minnesota (blue). (**d)** Mean annual precipitation (green; 9-year running mean in black) for Kenora and Sudbury.

**Figure 5:**
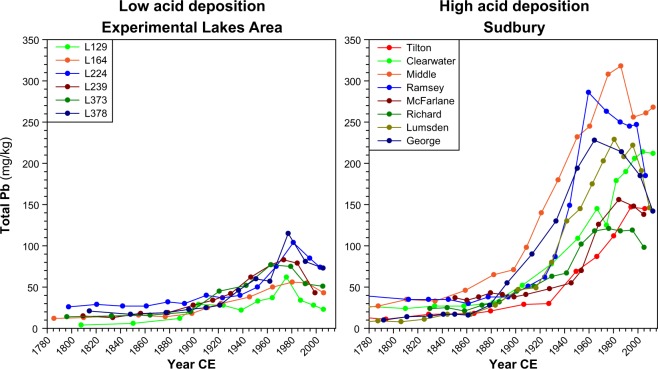
Total Pb concentrations (used here as a proxy for changes in atmospheric pollutant deposition, including sulphur, following industrialization) in the sediment records from the Experimental Lakes Area (ELA) and Sudbury, illustrating the difference in atmospheric deposition loads between the two regions.

Lake-water DOC concentrations inferred from the lake sediments are generally overestimated for the study lakes (Supplementary Fig. S3), but inferred trends closely match trends in monitoring data in both regions (Supplementary Fig. S4). Therefore, we focus on discussing overall trends and trajectories of change using our methods^26^. For Sudbury lakes, the DOC reconstructions show homogeneous trends, with stable levels until ~1850, declines beginning in ~1890, reaching their lowest values in the ~1960s, and increasing from the ~1970s/1980s (Figs 3 and 4). Compared to pre-industrial levels, DOC concentrations declined on average by 46 ± 14% (range: 27.6–63.4%) by the 1960s, and were still 29 ± 6% (range: 20.8–40.3%) below background levels by the 2000s (Fig. 4, Supplementary Fig. S6). These dynamics closely mirror temporal changes in acid deposition at the study sites, indicated by coeval changes in sediment total Pb concentrations (a robust proxy for changes in atmospheric pollutant deposition, including sulphur, following industrialization; Fig. 5), and in SO_2_ emissions for Sudbury and North America overall^30^ (Fig. 4), demonstrating the predominant influence of acid deposition on DOC dynamics in this former high deposition area. DOC concentrations started to decline shortly after the onset of local SO_2_ emissions following the discovery of nickel-copper sulphides (1883) and the establishment of open roast yards and the first smelters in 1888, and then began to recover in most lakes within two decades after 1970s emissions reductions. Vegetation loss following acid deposition and soil erosion in lake catchments located close to smelters may have contributed to the reduced DOC levels. However, two of the eight Sudbury lakes are located in Killarney Provincial Park, an area with intact vegetation cover ~60 km SW of Sudbury. DOC reconstructions for these lakes show a similar magnitude of change in DOC concentrations, pointing towards the overriding importance of acid deposition on soil DOC solubility/mobility rather than degradation of the terrestrial C pool. This is further corroborated by the ubiquitous measured DOC increases across the Sudbury region that include lakes with minimal catchment disturbances located far outside (up to 100 km) of the vegetation damage zone close to Sudbury smelters^25^. Elevated DOC levels in some lakes around 1870 coincide with initial logging activities in the area, which may have increased DOC leaching from forest soils over the short term^31,32^.

**Figure 6:**
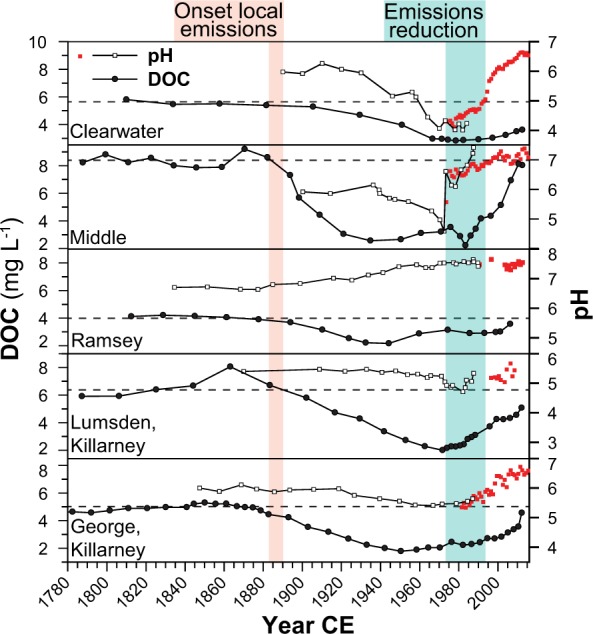
Changes in lake-water dissolved organic carbon (DOC) compared to different lake acidification histories, demonstrating the near-immediate response in DOC levels to changes in atmospheric acid deposition independent of lake-water acidification. Open squares (pH) and black circles (DOC) represent sediment-inferred trends, while red squares represent pH monitoring data. Historical pH trends were inferred for five of the eight Sudbury lakes in prior studies using diatom-based pH models^34,58–60^.

Sudbury lakes show variable acidification and recovery histories because of differences in catchment geology and thus differing acid buffering capacities^33^ (Ca^2+^ + Mg^2+^ concentration (CaMg*, indicating lake/catchment sensitivity to acidification[1]) = 32–372 µeq L^−1^; Supplementary Table S1), or because of direct management actions (e.g., liming in Middle Lake), whereby some lakes did not acidify or acidified at different points in time during the 20^th^ century (1920s to 1940s; Fig. 6, Supplementary Fig. S1). Despite different acidification histories, we observe the same DOC trajectories in all lakes, with a near-immediate response to changes in acid deposition (Figs 4 and 6), showing the dominance of direct physicochemical changes in soil-solution chemistry rather than in-lake processes for the observed lake-water DOC changes. The largest relative DOC declines (>60%) occurred in Middle Lake, located closest to Sudbury smelters (~5 km; no CaMg* measurements prior to liming) as well as in the most acid-sensitive lakes (George and Lumsden lakes, ~60 km distance to nearest smelters; CaMg* = 32–50 µeq L^−1^; Supplementary Table S1, Figs S5 and S6), exemplifying the importance of deposition load and catchment sensitivity to acidification for the scale of DOC change.

In general, increasing DOC trends are mainly observed in acid-sensitive regions^1,2^. Our sediment-inferred long-term trends also show pronounced DOC changes in three well-buffered central Sudbury lakes that likely never acidified^33,34^ (Ramsey, McFarlane and Richard lakes; 8–10 km distance to nearest smelter; CaMg* = 242–372 µeq L^−1^; Figs 3 and 6; Supplementary Table S1, Figs S1, S5 and S6). Similarly, significantly increasing stream-water DOC levels have been shown for a base-rich and well-buffered catchment in central Europe^19^. Previous studies have identified two potential physicochemical mechanisms for increasing DOC in response to reductions in acid deposition: a) reduced soil-solution acidity and/or b) reduced soil-solution ionic strength. Reduced acidity decreases the protonation of organic acids (e.g., carboxylic acids), which increases the electrical charge, and consequently the solubility and mobility of soil organic matter, whereas reduced ionic strength, more precisely reduced concentrations of polyvalent cations such as Al^3+^, Ca^2+^ and Mg^2+^, decreases the potential for DOC adsorption or coagulation through cation bridging^35–37^. As a consequence of reductions in acid deposition, substantial declines in polyvalent base cations also occurred in Sudbury lakes^25^ (Supplementary Fig. S5). The similar DOC response in all lakes independent of their individual catchment sensitivities to acidification suggests that changes in soil solution acidity and ionic strength are important for DOC dynamics in the Sudbury region, pointing towards a universal impact of acid deposition on lake-water DOC in both acid-sensitive and well-buffered systems^19^.

Similar to Sudbury, DOC concentrations were stable in the more remote ELA lakes prior to industrialization but also started to decline during the ~1890s with increasing SO_2_ emissions in North America and local SO_4_^2−^ deposition from long-range transport (Figs 3 and 4). The inferred DOC decline also coincides in ELA lakes with the rise of sediment total Pb concentrations, emphasizing the link between acid deposition and changing DOC levels (Fig. 5). By the 1920s, DOC concentrations declined on average by 11 ± 6% (range: 0.3–18%) relative to background levels, whereas in Sudbury average DOC values were already 33 ± 15% (range: 16–63%) below background (Fig. 4, Supplementary Fig. S6), reflecting the higher deposition rates in northeastern Ontario (Figs 1 and 2). In the ELA, catchment sensitivity of lakes to acidification (CaMg* = 75–199 µeq L^−1^) is comparable to Sudbury lakes, and ranges between those of the acid-sensitive (32–85 µeq L^−1^) and well-buffered (242–372 µeq L^−1^) Sudbury lakes (Supplementary Table S1).

From the 1930s onwards, however, DOC trends in ELA lakes differ from Sudbury lakes, gradually increasing to pre-industrial levels by the 1970s and then exceeding background levels by 11 ± 14% (range: −11.4 to +35.2%) in the 2000s (Fig. 4, Supplementary Fig. S6). If acid deposition was the sole stressor in the ELA, DOC concentrations would have remained suppressed until at least the 1970s. There are no indications of significant land use/cover changes during the 1900s in the ELA, which was established in 1968 in a wilderness area, with some logging in the 1950–1970s. Wildfires occur in the area every 80–120 years, reflected in forest stands of similar age^38^. Other stressors with a potential to elevate DOC levels in the ELA are changes in climate and atmospheric N deposition. Increased N deposition can promote plant growth in N-limited forests, which increases the C pool and thus the potential for DOC leaching^15,16^. In the ELA, N deposition likely increased over the past century with increasing fuel combustion and agricultural activities but shows no significant trend since the beginning of monitoring in ~1970 (Fig. 2)^38,39^.

Significant changes in climate, however, occurred over the past century in the ELA, with increased rainfall by 160 mm and ~1.7 °C warmer mean annual air temperatures, prolonging the main growing and runoff season by ~17 days (i.e., days with temperatures above 0 °C) (Fig. 2). Warmer temperatures, increased vegetation cover and changes in hydrology have been linked to elevated DOC levels in freshwaters^13,17,18^, and may explain the increasing DOC trend in ELA lakes since the 1930s. Although monitoring data show that these climate changes occurred mainly since the 1970s, the nearest longer-term meteorological record (Fort Snelling, Minnesota, Fig. 1)^40^ suggests that temperatures in central North America were, on average, already warmer since the 1930s compared to the preceding 100 years (Fig. 4). An earlier change of environmental conditions is supported by analyses of historical biological communities (diatoms, chrysophytes) in the study lakes, which indicate changing lake thermal properties (increasing thermal stability, warmer surface-water temperatures, shorter ice-covered season) and possibly increased DOC concentrations since the early-1900s^41,42^.

Acid deposition was the dominant stressor affecting lake-water DOC levels in Sudbury and likely other higher deposition areas over the past century, leading to the current widespread re-browning of lakes with reduced acid inputs. Compared to acid deposition, the relative impact of climate change on DOC concentrations appears to be considerably lower. However, in the ELA the current DOC increase has already surpassed pre-industrial background levels in most lakes, suggesting that climate is already the dominant driver of DOC change in low acid-deposition regions. In the past, the Sudbury region experienced similar environmental changes (increased annual air temperature by ~1.4 °C, increased rainfall by ~145 mm, and a longer growing and runoff season by ~21 days since the 1950s; Fig. 2) as the ELA. Consequently, these changes will also have contributed to the ongoing DOC increase in Sudbury lakes over the last decades^43^. While we cannot differentiate between the co-occurring individual contributions of these environmental changes and reductions in acid deposition to the current DOC increase, our results from the low deposition ELA region suggest that the changing climate, together with the effects of acidification recovery, will lead to further increases in DOC concentrations, and also shift the post-acidification baseline above pre-industrial DOC levels in former high acid deposition regions. However, when identifying appropriate post-acidification DOC reference levels, particularly in more urban settings, it is important to also consider other human-induced environmental changes that have occurred since pre-industrial times that may affect the terrestrial and/or aquatic DOC supply (e.g., changes in vegetation cover and composition, infrastructure development, and lake eutrophication).

Previous assessments attempting to determine browning patterns in lakes were hampered by relatively short periods of environmental monitoring. Not surprisingly, conclusions tended to focus on the browning process itself, often concluding that lakes are entering new ecological states. Our data provide new insights into the scale of long-term DOC changes associated with different stressors relative to pre-industrial levels. In a multiple stressor world, alternate scenarios of browning and re-browning likely exist, with the latter more common to regions that received moderate to high levels of acid deposition. These widespread fundamental changes in lakes may have large consequences for the biota in these ecosystems, as well as management implications for fisheries and drinking-water resources.

## Methods

### Sediment sampling and dating

Sediment cores were recovered from the deepest basin of each lake in 2006 and 2012 for Sudbury/Killarney lakes and in 2003 and 2006 for ELA lakes using a gravity corer (Supplementary Table S1). All sediment cores were radiometrically dated by analysing ^210^Pb, ^226^Ra (via its granddaughter isotope ^214^Pb) and ^137^Cs using gamma spectrometry. Age-depth relationships for the past 100–150 years were calculated based on ^210^Pb activities and cumulative dry mass using the constant rate of ^210^Pb supply (CRS) dating model^44^. Sediment ages beyond the ^210^Pb dating range were estimated based on 2^nd^ order polynomial extrapolations of the ^210^Pb chronologies until ~1780. More specific information regarding lake and watershed characteristics and individual dating results are provided in earlier studies^33,42,45–49^.

### Sediment-inferred lake-water DOC

Past lake-water DOC concentrations were reconstructed by employing a transfer function between VNIR spectra of lake-surface sediments (i.e., the most recently accumulated material) and corresponding measured organic carbon concentrations in the surface water^26^. The partial least squares regression (PLSR) model is calibrated against lake-water total organic carbon (TOC) concentrations but we interpret these as virtually equivalent to DOC because DOC greatly dominates the TOC pool in lakes used for establishing the model as well as in our study lakes. For example, in our ELA lakes, TOC is on average composed of ~91% DOC, with an average particulate organic carbon content of ~0.7 mg L^−1^. The calibration model is based on 345 Arctic, boreal and northern temperate lakes from Canada, Greenland, Sweden and Finland, covers a TOC range from 0.5 to 41 mg L^−1^, and has a cross-validated R^2^ of 0.57 and a prediction error of 4.4 mg TOC L^−1 ^^26^. Prior to analyses, sediment samples were freeze-dried and sieved (125 μm mesh) to remove the effects of water and particle size on the VNIR signal. VNIR spectra of samples from the calibration set and the study lakes were recorded with a FOSS NIRSystem 6500 rapid content analyser (Hillerød, Denmark) in diffuse reflectance mode. Each sediment sample spectra represents a mean of 32 scans at 2 nm resolution in the wavelength range from 400 to 2500 nm. PLSR modelling and lake-water TOC reconstructions were performed using SIMCA 14.0 (Umetrics AB, Umeå, Sweden). The application of the method to annually laminated sediments that have repeatedly been sampled over 27 years demonstrated that sediment-based TOC reconstructions are not biased by post-depositional, diagenetic changes in sediment composition^26^. To minimize potential storage-related changes in sediment composition, sediment samples were stored protected from direct light exposure in sealed sample containers at all times.

The DOC inference model was developed with the explicit intention of reconstructing trends in past lake-water DOC concentrations. Although the model generally overestimates absolute values in the study lakes from Sudbury and the ELA, inferred and observed trends correspond closely (Supplementary Figs S3 and S4). For ease of comparison, we standardized inferred long-term DOC trends to illustrate the model’s ability to accurately reconstruct relative DOC changes in the study lakes. Regional relative DOC trends for Sudbury and the ELA were established based on mean DOC concentration changes relative to pre-industrial DOC levels (1780–1850) for the eight lakes from each region. To assure equal weight of each record to the regional trend, temporal DOC changes in each lake were binned (20-year bins for the period 1780–1860 and 10-year bins from 1860 onwards) and missing intervals were interpolated from adjacent intervals by linear regression (Supplementary Fig. S6).

### Water chemistry, climate and deposition data

The study lakes are part of monitoring programs led by the International Institute for Sustainable Development for the ELA and the Ontario Ministry of the Environment, Conservation and Parks for Sudbury. Water-chemistry data are presented as annual epilimnetic means for the open-water season (May to October) (Supplementary Figs S1–3 and 5). Annual atmospheric bulk SO_4_^2−^ and TN deposition were calculated as products of the mean SO_4_^2−^ and TN concentration in precipitation and the amount of precipitation (Fig. 2). Temperature and precipitation data were retrieved from Environment and Climate Change Canada using the second generation of the adjusted and homogenized Canadian climate data, with data for Kenora from 1900 to 2012 and for Sudbury from 1922 to 2012 for precipitation and from 1948 to 2012 for temperature^50^. The long-term temperature record for Minnesota, based on measurements from Fort Snelling (1820–1982)^40^, was extended to 2017 by using temperature data from the nearby Minneapolis-Saint Paul airport. The temperature anomaly presented in Fig. 4 is based on mean annual temperatures relative to the average annual temperature over the period 1820–2017. Smoothed trends are based on 9-year running means for all variables. We used the Mann-Kendall test on annual mean data to determine significant long-term trends (p < 0.05) in climate, deposition and water-chemistry data for our study lakes with at least 25 years of monitoring data (ELA: n = 3; Sudbury/Killarney: n = 4). The Theil-Sen slope estimator was used to quantify annual temporal changes, and relative changes in DOC concentrations are based on Theil-Sen slopes relative to mean DOC levels for the monitoring period. All statistical tests were performed in the R environment^51^ using the ‘Kendall’^52^ and ‘zyp’ package^53^, respectively.

### Total Pb

Given that sulphur can be mobile in sediments^54^, total Pb concentrations were used as an indicator of the timing and level of atmospheric pollutant deposition in Sudbury/Killarney and the ELA (Fig. 5). Pb emissions increased in a similar manner to sulphur emissions following industrialization as a consequence of increased ore smelting, combustion of coal and, later, leaded gasoline, which peaked in the 1970s^30,55^. Pb concentrations were determined on freeze-dried powdered sample material by wavelength dispersive X-ray fluorescence using a Bruker S8 Tiger spectrometer^56^ in all sediment cores from Sudbury and in six out of eight sediment cores from the ELA.

## Supplementary information

Supplementary Information

## Data Availability

All data generated or analysed during this study are included in this published article and its Supplementary Information files, and spectroscopic datasets generated during the current study are available from the corresponding author on reasonable request.
