# Supplementary material for: The browning and re-browning of lakes: Divergent lake-water organic carbon trends linked to acid deposition and climate change
Source: Sci Rep. 2019 Nov 13;9:16676. doi: 10.1038/s41598-019-52912-0 (PMC6853936; doi:10.1038/s41598-019-52912-0)
Supplement: Supplementary file 1 — Supplementary Information [file 41598_2019_52912_MOESM1_ESM.pdf]

**Supplementary to "The browning and re-browning of lakes: Divergent lake-water organic carbon trends linked to acid deposition and climate change"**

*Carsten Meyer-Jacob*[*,1,2], *Neal Michelutti*[1], *Andrew M. Paterson*[3], *Brian F. Cumming*[1], *Wendel (Bill) Keller*[4] & *John P. Smol*[1]

[1] Paleoecological Environmental Assessment and Research Laboratory (PEARL), Department of Biology, Queen's University, Kingston, ON K7L 3N6, Canada

[2] Department of Ecology and Environmental Science, Umeå University, 90187 Umeå, Sweden.

[3] Dorset Environmental Science Centre, Ontario Ministry of the Environment and Climate Change, Dorset, ON P0A 1E0, Canada

[4] Cooperative Freshwater Ecology Unit, Vale Living with Lakes Centre, Laurentian University, Sudbury, ON P3E 2C6, Canada

8 pages, 1 table, 6 figures

**Table S1.** Location, morphometric characteristics and selected water chemistry data (mean of available data for the period 2005 to 2015 (May-October)) for the study lakes.

| **Lake** | **Coordinates** | **Elevation** (m a.s.l.) | **Lake area** (ha) | $Z_{max}$ (m) | $Z_{mean}$ (m) | **DOC** (mg $L^{-1}$) | **pH** | **TP** (µg $L^{-1}$) | $SO_4^{2-}$ (mg $L^{-1}$) | **CaMg*** (µeq $L^{-1}$) |
|---|---|---|---|---|---|---|---|---|---|---|
| **Sudbury** | | | | | | | | | | |
| Tilton | 46°21.40 N, 81°04.30 W | 252 | 52 | 12 | 7 | 2.9 | 6.8 | 5.6 | 7.5 | 77 |
| Clearwater | 46°22.20 N, 81°03.04 W | 267 | 76 | 22 | 8 | 3.0 | 6.5 | 4.1 | 8.7 | 85 |
| Middle | 46°26.34 N, 81°01.44 W | 278 | 28 | 15 | 6 | 4.0 | 7.1 | 6.9 | 15.4 | 270[1] |
| Ramsey | 46°28.60 N, 80°56.90 W | 251 | 795 | 21 | 8 | 3.6 | 7.5 | 10.3 | 17.3 | 372 |
| McFarlane | 46°24.85 N, 80°57.85 W | 226 | 166 | 18 | 7 | 4.4 | 7.6 | 9.0 | 17.5 | 361 |
| Richard | 46°26.25 N, 80°55.00 W | 258 | 84 | 9 | 4 | 3.1 | 7.3 | 9.6 | 8.4 | 242 |
| **Killarney Provincial Park** | | | | | | | | | | |
| Lumsden | 46°01.52 N, 81°25.97 W | 238 | 22 | 22 | 7 | 1.4 | 5.5 | 2.8 | 5.2 | 32 |
| George | 46°01.79 N, 81°23.83 W | 189 | 189 | 40 | 19 | 2.1 | 6.5 | 3.6 | 5.7 | 50 |
| **Experimental Lakes Area** | | | | | | | | | | |
| L127 | 49°38.47 N, 93°51.30 W | 427 | 5 | 5 | 2 | 5.7 | 6.8 | 7.6 | 2.0 | 75 |
| L129 | 49°38.67 N, 93°50.12 W | 406 | 5 | 5 | 2 | 9.6 | 6.5 | 8.3 | 1.6 | 114 |
| L164 | 49°37.25 N, 93°49.20 W | 369 | 20 | 7 | 5 | 8.8 | 6.7 | 5.1 | 1.5 | 136 |
| L224 | 49°41.40 N, 93°43.00 W | 408 | 26 | 27 | 12 | 3.6 | 7.0 | 2.2 | 1.9 | 117 |
| L239 | 49°39.75 N, 93°43.35 W | 386 | 54 | 32 | 11 | 7.2 | 7.1 | 2.3 | 2.4 | 199 |
| L373 | 49°44.65 N, 93°47.98 W | 424 | 27 | 21 | 11 | 4.2 | 7.2 | 3.5 | 1.9 | 191 |
| L377 | 49°43.25 N, 93°46.42 W | 392 | 27 | 18 | 9 | 5.5 | 7.0 | 4.0 | 2.3 | 163 |
| L378 | 49°42.64 N, 93°46.58 W | 396 | 24 | 18 | 7 | 7.4 | 6.9 | 2.3 | 1.9 | 140 |

Abbreviations: maximum lake depth ($Z_{max}$), mean lake depth ($Z_{mean}$), dissolved organic carbon (DOC), total phosphorus (TP), sulphate ($SO_4^{2-}$), and calcium ($Ca^{2+}$) + magnesium ($Mg^{2+}$) concentrations (CaMg*). [1]Middle Lake was limed in 1973 prior to the beginning of water-chemistry monitoring.

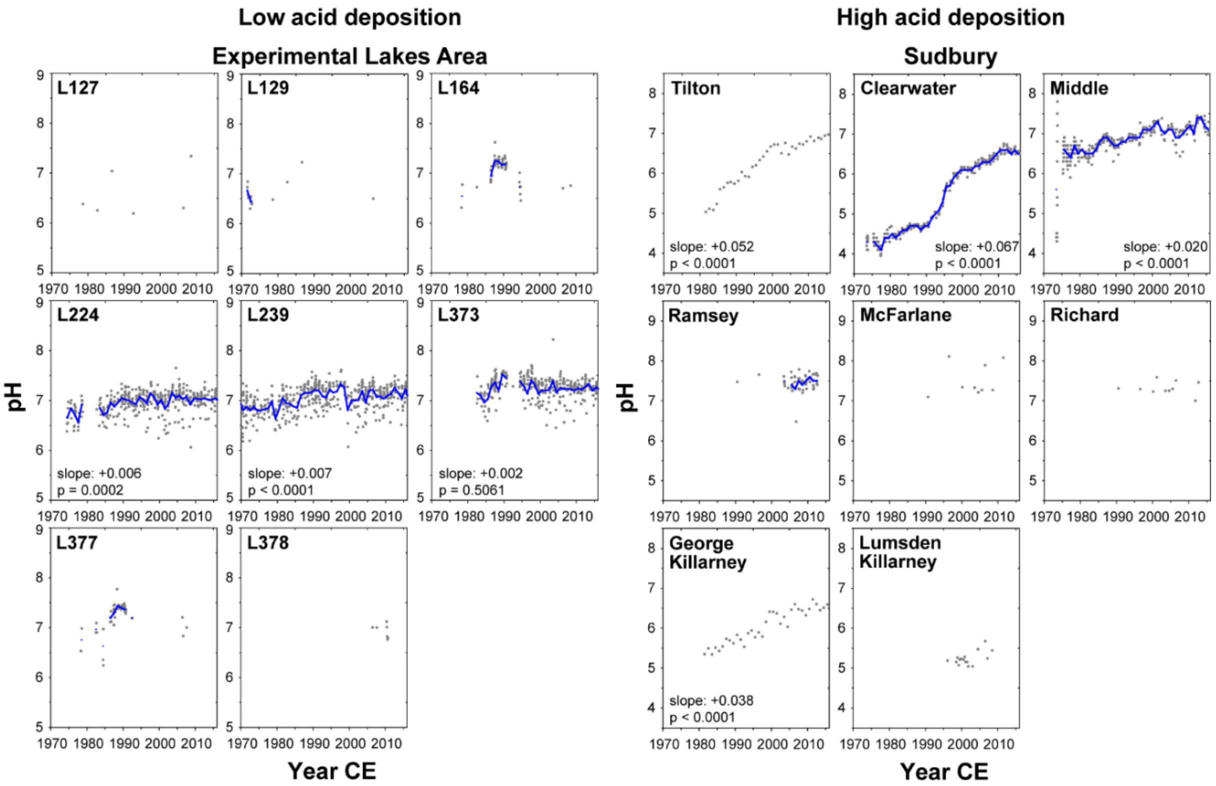


**Figure S1.** pH monitoring data for the study lakes. Grey circles represent individual measurements, while blue lines represent annual means. Slopes (annual change) and significance levels resulting from trend analyses are given for records with at least 25 years of data.

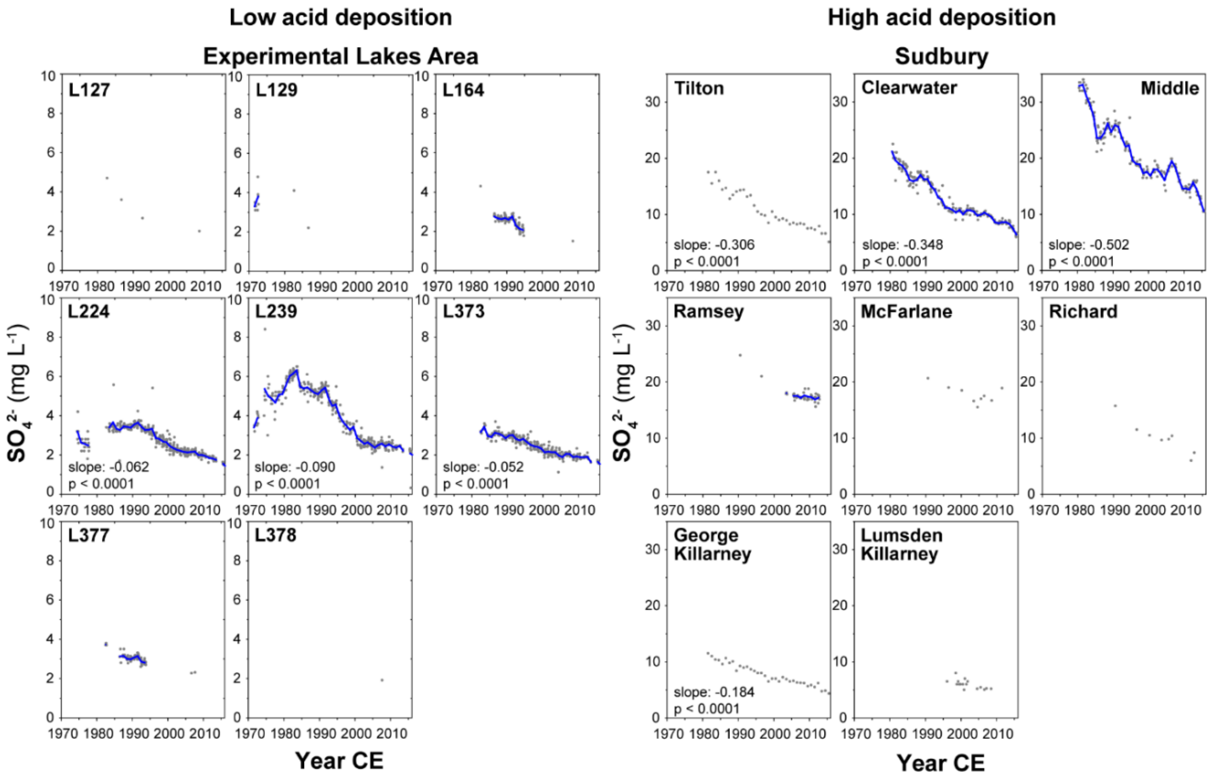


**Figure S2.** Sulphate ($SO_4^{2-}$) monitoring data for the study lakes. Grey circles represent individual measurements, while blue lines represent annual means. Slopes (annual change) and significance levels resulting from trend analyses are given for records with at least 25 years of data.

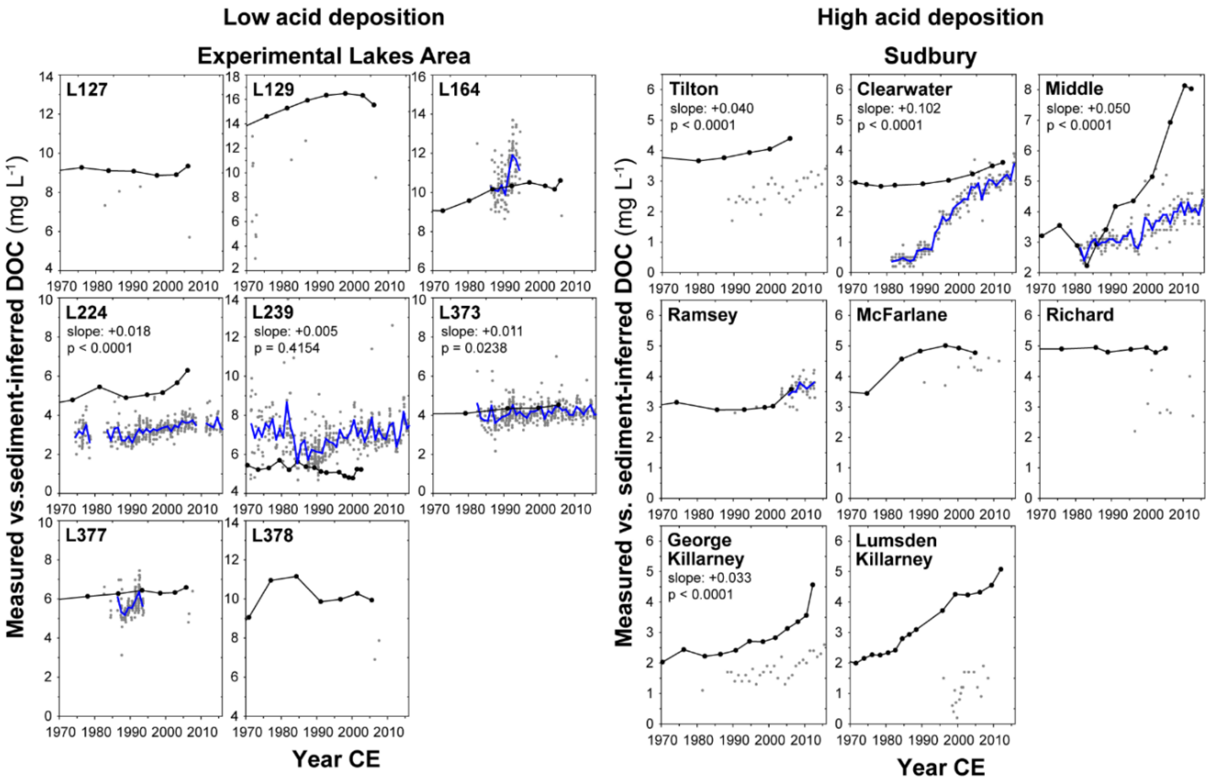


**Figure S3.** Monitored (grey circles; annual mean – blue line plot) versus sediment-inferred (black circles) lake-water dissolved organic carbon (DOC) concentrations for the study lakes. Slopes (annual change) and significance levels resulting from trend analyses are given for records with at least 25 years of monitoring data.

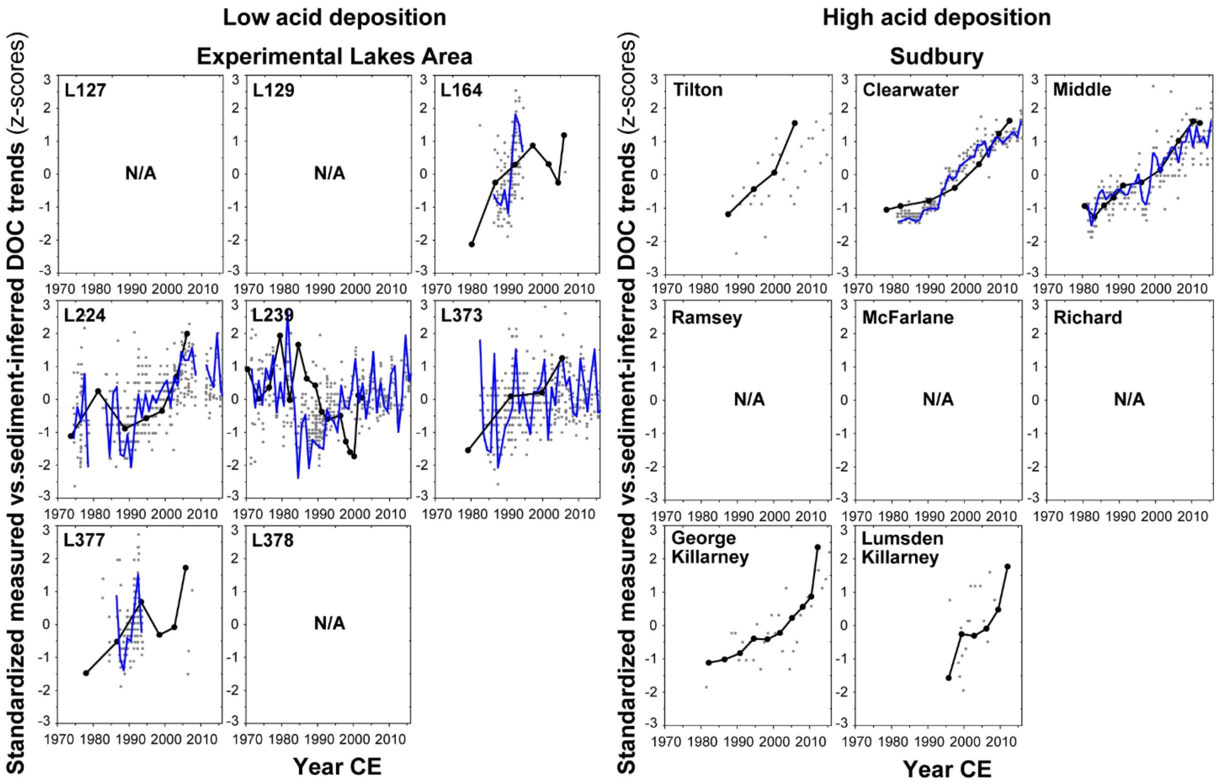


**Figure S4.** Comparison of standardized (z-scores) monitored (grey circles; annual mean – blue line plot) and sediment-inferred (black circles) lake-water dissolved organic carbon (DOC) trends for study lakes with at least 10 years of overlapping monitoring and sediment-inferred data.

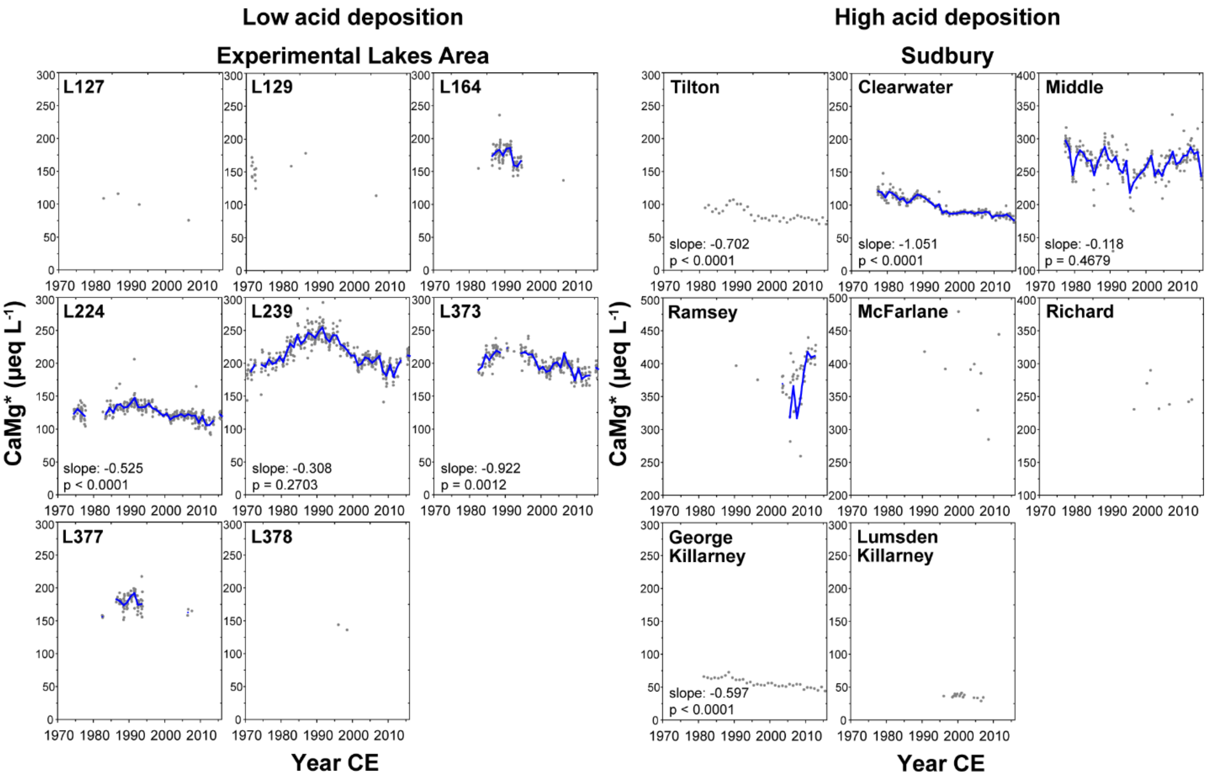


**Figure S5.** Concentrations of $Ca^{2+}$ and $Mg^{2+}$ (CaMg*) monitoring data for the study lakes. Grey circles represent individual measurements, while blue lines represent annual means. Slopes (annual change) and significance levels resulting from trend analyses are given for records with at least 25 years of data.

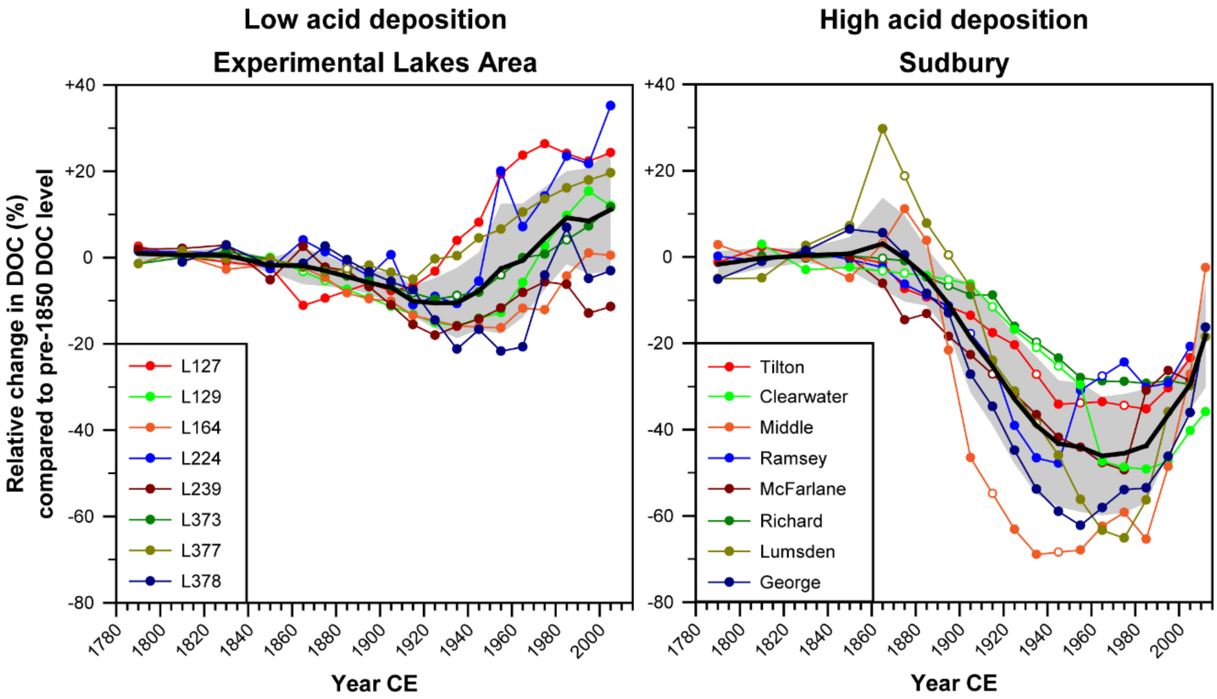


**Figure S6.** Relative change in sediment-inferred dissolved organic carbon (DOC) compared to the pre-industrial (1780–1850) mean DOC concentration. Coloured circles represent binned DOC reconstructions in 20- (1780–1860) and 10-year intervals (from 1860 onwards) for the individual lakes (open circles indicate bins with interpolated data), while the black line plot and the grey envelope represent the regional DOC trend and its standard deviation for the Experimental Lakes Area and Sudbury, based on the eight individual records for each region.
